# How Cortisol Reactivity Influences Prosocial Decision-Making: The Moderating Role of Sex and Empathic Concern

**DOI:** 10.3389/fnhum.2019.00415

**Published:** 2019-11-26

**Authors:** Qionghan Zhang, Jianhong Ma, Urs M. Nater

**Affiliations:** ^1^Department of Psychology and Behavioral Sciences, Zhejiang University, Hangzhou, China; ^2^Department of Psychology, Philipps-University of Marburg, Marburg, Germany; ^3^Department of Clinical Psychology, University of Vienna, Vienna, Austria

**Keywords:** cortisol, decision-making, empathic concern, sex difference, stress, tend-and-befriend

## Abstract

The fight and flight theory and the tend-and-befriend theory suggest two opposite behavioral stress responses, and heterogeneous research results revealed the importance of taking sex into account. The experiment was designed to investigate the effect of stress-related cortisol reactivity on subsequent prosocial decision-making behaviors, and the moderating role of sex and empathic concern (EC) in the process. Sixty-one healthy students (34 women, 27 men) underwent the Trier Social Stress Test for Groups (TSST-G) or the control condition. Subsequently, participants completed three economic tasks—the dictator game, the ultimatum game, and the third-party compensation game. Statistical analyses revealed a significant main effect of cortisol reactivity on individuals’ third-party compensation behaviorssex. A sex-specific effect of stress-related cortisol change on prosocial behaviors was found, with men behaving more generously in the dictator game as stress-related cortisol reactivity increased. Furthermore, the level of EC was found to moderate the association between stress-related cortisol change and prosocial behaviors, that individuals with a low level of EC reported more generosity and third-party compensation behaviors. Overall, the present study contributes to a better understanding of the behavioral stress responses, that individuals whose hypothalamic-pituitary-adrenal (HPA) axis are highly activated in response to stress would exhibit tend-and-befriend responses, but only among men and those with a low level of EC.

## Introduction

When faced with a stressful situation, the human body will adjust itself to maintain stability, which is defined as allostasis (McEwen, 1998). The process of adjustment results in a quick response of the autonomic nervous system (ANS) and a slower response of hypothalamic-pituitary-adrenal (HPA) with its final output product cortisol (Skoluda et al., 2015). Several studies have suggested that the cortisol reactivity to stress may modulate behavioral responses to stress, such as decision-making behaviors in the context of social interactions (Akinola and Mendes, 2012; Starcke and Brand, 2012; Kimura et al., 2013). In terms of behavioral responses to psychological stress, humans are believed to build adaptive reactions to stress gradually. The fight-or-flight theory (Cannon, 1932), which suggests that individuals tend to care more about their interests, hence making them less prosocial when stressed, has been the dominant conception for decades. Some scholars criticized this theory because it developed from research with only male participants, and thus doubts were gradually raised regarding its applicability to women. Therefore, the tend-and-befriend theory emerged and posited that stressed women have a desire to tend to children, affiliate with others and engage in prosocial behaviors to promote survival (Taylor et al., 2000; Taylor, 2006). While accumulating evidence have supported the tend-and-befriend theory among women (Tomova et al., 2014; von Dawans et al., 2019), Geary and Flinn (2002) argued that the nature of parental investment rooted in both genders, and befriending could also be a consequence of men’s fight-or-flight response to stress. Some studies supported their perspective that tend-and-befriend theory is not exclusive to women, and stressed men also exhibited prosocial or affiliative behaviors (Youssef et al., 2012; von Dawans et al., 2012; Margittai et al., 2015; Berger et al., 2016). An alternative theory suggested that it is a universal pattern that individuals engage in prosocial behaviors to mitigate the negative effects due to stress and maintain the mental health (Aknin et al., 2013; Raposa et al., 2016).

On the basis of studies about humans’ behavioral responses to stress, recent findings have gradually explored the influence of stress on human’s prosocial decision-making behaviors, and the findings were diverse when sex was taken into account. For instance, a study used a one-shot variant of the dictator game to assess general altruism, in which a participant decided how much money (0–10€) to donate to a charitable organization anonymously while keeping the rest (Vinkers et al., 2013). The researchers found that compared to men in the control group, men in the stress group were less willing to donate in the dictator game. In contrast, in another study measuring decision-making behaviors following stress, men showed higher scores on trust, trustworthiness and made more divisions to a stranger in the dictator game following the group version of the Trier Social Stress Test (TSST-G; von Dawans et al., 2011) compared to a control group (von Dawans et al., 2012). Numerous studies have demonstrated an increase in prosocial behaviors of women under acute stress (Smeets et al., 2009; Tomova et al., 2014). As an example, acute stress was shown to induce women’s prosocial trustworthiness and sharing behaviors (von Dawans et al., 2019). In another recent study including both genders, the influences of stress on individuals’ second party punishment behavior (also known as altruistic punishment behavior), cooperation and prosocial risk-taking were examined (Nickels et al., 2017). The majority of the results supported the sexually dimorphic hypothesis of the fight-or-flight theory and tend-and-befriend theory (i.e., stressed men more selfish and less prosocial while stressed women more generous and cooperative), however, there were some inconsistent findings that stress has no influence on men’s cooperative behaviors in the prisoner’s dilemma, or women’s prosocial risk-taking behaviors. These mixed findings illustrate that the important role of sex should be considered when examining individuals’ decision-making processes under acute stress.

Apart from merely comparing the differences of individuals’ behaviors between the stress and control group, several studies further explored the impact of stress-induced cortisol elevation on decision-making behaviors, but failed to consider the role of sex. For example, Singer et al. (2017) found positive associations between the magnitude of cortisol level and men’s altruistic decision-making behaviors, but women were not included as participants. In a study that failed to observe a significant difference in men’s decision-making between the stress group and control group, the authors found a negative correlation between cortisol reactivity and altruistic decisions in the high-emotional moral dilemmas (Starcke et al., 2011). In another study, no association between stress-induced cortisol change and prosocial decision-making behaviors of men was observed by von Dawans et al. (2012), even though they did report a significant difference in prosocial behaviors (i.e., trust, trustworthiness, and sharing behaviors) between a stress group and a control group. Although von Dawans et al. (2019) later detected that women engaged more trustworthy and sharing behaviors in the stress group compared to the control group, they did not further consider the relationship between cortisol elevation and decision-making behaviors. Thus, the first objective of the present study was to determine the role of sex in the relationship between the cortisol change to stress and decision-making behaviors immediately after stress.

The above findings concerning potential sex differences of behavioral stress responses challenged the traditional state-dependent perspectives which stated that powerful situations such as stress might produce consistent behavioral consequences regardless of individual differences (Milgram, 1963; Block, 1968). Some research integrated the state-dependent hypotheses with the trait-consistent assumptions (Epstein, 1983; Kenrick and Funder, 1988), and suggested a possible interactive effect of trait and situation on human behaviors (Mischel and Shoda, 1995; Cooper and Withey, 2009; Finy et al., 2014). When it comes to social decision-making behavior, the importance of trait empathy can never be overemphasized, as well as its interaction with stress and stress-induced cortisol change.

Trait empathy, or empathic concern (EC), also known as affective empathy (Edele et al., 2013), reflects feelings of concern for individuals suffering from sad circumstances (Davis, 1983). Previous studies have established that trait empathy predicts prosocial behaviors in different social decision tasks, such as the ultimatum game (Barraza and Zak, 2009), the dictator game (Edele et al., 2013), and the third-party compensation game (Leliveld et al., 2012; Hu et al., 2015). When Hiraoka and Nomura (2017) examined the influence of stress on women’s response to infant crying after the Trier Social Stress Test (TSST), EC was found to modulate the process. Although women’s care intentions are not identical to the prosocial decision-making behaviors examined in the present study, the pattern supports the concept that the level of EC may play a role in modulating individuals’ decision-making behavioral responses to stress. Hence, the second objective of the current research is to explore the role of EC in prosocial decision-making behaviors immediately after stress, as well as its interactive effect with cortisol reactivity.

The integrated theory of state-dependent and trait-consistent assumptions believed that certain traits can be consistently predicted on human behaviors across different situations, while certain situations can have a consistent influence on humans’ behaviors with different levels of traits. For individuals with high EC who have strong concerns towards others, it is natural for them to show high prosocial behaviors regardless of the situations, and hence, a stressful situation or stress-induced cortisol change may have weaker impacts on their prosocial behaviors. On the opposite, individuals with low EC exhibited low prosocial behaviors in neutral situations, but their prosocial behaviors may be strongly affected by stressors or stress-related cortisol change because they are not good at coping with stress. This is based on one research that linked dispositional low empathy (narcissism) to higher elevated cortisol responses to stressors (Edelstein et al., 2010). In short, this study hypothesized that stress and stress-related cortisol reactivity were more predictive on decision-making behaviors at a low level of EC, but not on the high level of EC.

## Materials and Methods

### Participants

We recruited medically healthy students who were enrolled at the University of Marburg through the university mailing list. Potential subjects were screened in a telephone interview according to the following exclusion criteria: lack of fluency in the German language, age <18 or >35, pregnancy or current breastfeeding, body mass index (BMI) >30 kg/m^2^, smoking >5 cigarettes/day, regular recreational drug use (cannabis consumption >2 weeks and others >1 year), chronic physical illness, intake of medication targeting the HPA axis, and previous participation in studies using the (TSST; Kirschbaum et al., 1993) including the group version (TSST-G) or in studies employing economic decision-making paradigms. Most of the female students at this age were using oral contraceptives and reported irregular menstruation. Following van den Bos et al. (2009), we decided not to take oral contraceptives and menstrual cycle into account. Participants who expressed doubts regarding the reality of the decision-making tasks (three persons), or had problems understanding the rules of the experiments (two persons), were also excluded from the analyses. This resulted in a final sample of 61 healthy students, who were assigned either to the control condition (*n* = 30, 20 women, 10 men) or the stress condition (*n* = 31, 14 women, 17 men). Assuming a medium effect size *f*^2^ = 0.15 (see Probst et al., 2017; Sherman et al., 2017), the sample size necessary to achieve a power of 0.8 and *α* = 0.05 was *N* = 55 for both the main effects and two-way interaction effect, calculated by *a priori* power analysis using the statistical software G-Power (Faul et al., 2007). As a result, the final sample size *N* = 61 has exceeded the minimum requirement to reach a reasonable conclusion.

Participants were told that they would be remunerated with 16€–20€ for their participation depending on their performance in the experiment, and would also have the opportunity to be entered into a lottery to win 50€. In fact, all participants received a maximum of 20€ at the end of the experiment and were entered into the lottery. All participants provided written informed consent. The study protocol was approved by the local Ethics Committee of the Department of Psychology at the University of Marburg, Germany.

### Social Decision-Making Paradigms

The current study adopted the commonly used dictator game (Takahashi et al., 2007; Leliveld et al., 2012; von Dawans et al., 2012; Vinkers et al., 2013) and the ultimatum game (Nickels et al., 2017; Youssef et al., 2018) for the assessment of the generosity and second-party punishment (2PP) behavior, respectively. For the exclusion of the potential revenge motivation underlying the 2PP behavior (Strobel et al., 2011; Youssef et al., 2018), the current research included the third-party compensation game for the measurement of participants’ pure prosocial behaviors towards a third party who suffered an unfair treatment, known as the third-party compensation (3PC) behaviors (Leliveld et al., 2012). The three social decision-making paradigms were programmed using software called “z-Tree” (Fischbacher, 2007), and consisted of two rounds of the ultimatum game, two rounds of the dictator game, and one round of the third-party compensation game. The order of the games was randomized. To ensure everyone played the decision-making games anonymously, participants in every game were only informed about the roles they were assigned, and that the other roles were assigned to other participants, but were not informed of any personal information about the other players.

#### Ultimatum Game

The ultimatum game consists of two parties, a proposer (player A) and a responder (player B). Each time, player A received 100 tokens (1 token = 0.05 Euro) and had to decide on how to allocate these tokens between the two players (offers had to be in multiples of 10 tokens). Player B was then asked to choose to either accept or reject the offer. If player B accepted the offer, both players received the money according to the offer, but if player B chose to reject, both players received nothing. To detect the lowest offer that player B would accept, we implemented the strategy method (Henrich et al., 2006) for the decision of player B: player B had to indicate his/her decision towards six possible proposals of player A (0, 10, 20, 30, 40, 50 tokens). The hypothetical decisions were binding because participants were informed that they were subsequently matched with player A’s actual decision and determined the outcome. All participants played the ultimatum game twice, once as the proposer (player A) and once as the responder (player B) in random order. The dependent variable was the lowest offer that player B would accept, as the assessment of 2PP behavior.

#### Dictator Game

The dictator game also consisted of a proposer (player A) and a responder (player B). Each time, player A received 100 tokens and decided on how to allocate these tokens between the two players. Player B had no choice but to accept whatever amount of tokens player A proposed to share with him/her. All participants played the dictator game twice, once as the proposer (player A) and once as the responder (player B) in random order. The dependent variable was the amount of tokens player A decided to allocate to player B, as the assessment of participants’ generosity in the dictator game.

#### Third-Party Compensation Game

Following the paradigm of the third-party compensation game (Leliveld et al., 2012), the present game involved three parties: player C observed the dictator game played by player A and player B. Player A made the decision to share 100 tokens with player B (offers had to be in multiples of 10 tokens), and player B had to accept the offer. Player C had an endowment of 50 tokens, and had to decide whether he/she would like to compensate player B. Every token player C donated would be multiplied by three and transferred to player B. The strategy method was also adopted here: player C had to indicate his/her decision towards six possible proposals of player A (0, 10, 20, 30, 40, 50 tokens). The hypothetical decisions of player C could reflect participants’ real decisions because they were told that these decisions would be subsequently matched with the actual decision of player A, and thus generate every player’s outcome. Participants played the third-party compensation game once as player C for the assessment of their third-party compensation behaviors.

### Stress Induction and Control Condition

The TSST-G (von Dawans et al., 2011) is a widely used, standardized task that contains elements of social-evaluative threat and uncontrollability while meeting the requirement of simultaneous stress induction for groups. In the first phase of the TSST-G stress task, participants were provided with instructions to prepare for an upcoming fictitious job interview (duration:10 min). Two research assistants in white coats were presented and evaluated participants for the next two phases. They were trained to refrain from providing any positive feedback and pretended to turn on a camera for recording. In the second phase of TSST-G stress task, each participant was asked to give a 2-min job interview speech for their dream job (duration: 8 min). In the final part of the TSST-G stress task, participants were randomly called upon several times to complete a mental arithmetic task (duration: 8 min).

Participants in the control group went through similar procedures but without any components related to social-evaluative threat or uncontrollability. In the first phase of the TSST-G control task, participants were instructed to read a scientific text for 10 min. Two research assistants were also presented in the test room for the next two phases, but they wore regular clothes, and were introduced only to provide necessary guidance for the tasks, but were not to observe or evaluate participants’ performances. No camera was used in the control condition. Participants read the scientific text out loud together in a quiet voice in the second phase of the task. In the final part of the TSST-G control task, participants completed an easy counting task.

### Procedure

**Figure 1:**
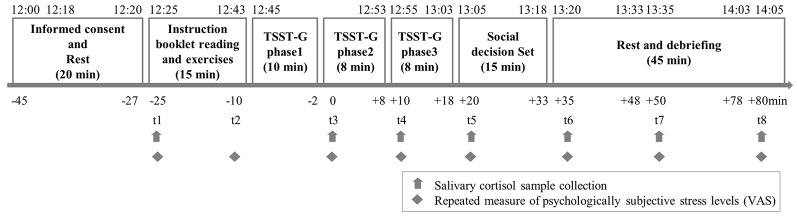
Study protocol, exact timing of each phases and the time of each phase relative to the start time of stress/control induction.

Five days before the experiment, participants completed an online questionnaire assessing the level of EC, major depression, dispositional stress reactivity, and chronic stress levels. The experimental session took place between 12:00 and 14:05 (see Figure 1) to control for the diurnal secretion pattern of cortisol. Participants were told not to do sports 24 h before the examination and to avoid physically strenuous activities 10 h before the appointment. They were not allowed to take chewing gum or smoke cigarettes for 24 h before the experiment, and to refrain from drinking alcohol and caffeinated beverages (such as coffee, soda, energy drinks) for 18 h before the examination. Furthermore, the participants were not allowed to eat or brush their teeth for at least 60 min before their arrival. For the group version of the TSST (TSST-G; von Dawans et al., 2011), participants were invited to the lab in groups of four and each group was randomly assigned to the stress or control condition.

Upon arrival at the laboratory, target participants were provided with informed consent forms to read and sign. They were then seated in a computer room separated by dividing walls and were asked not to communicate with each other for the whole experiment. After a 20-min resting period, they were asked to read the instruction booklet explaining the rules of three different decision-making games and completed some exercises to confirm their understanding of all the rules (duration: 15 min). Subsequently, participants either took part in the TSST-G stress task or a control task (duration: 26 min). The second and third phases of the TSST-G task (or control task) happened in a nearby test room, and all participants were guided in and out of the test room individually by the experimenter. When participants experienced the TSST-G task (or control task) together, they were also separated by dividing walls in the test room, so that all possible interactions between each other were restricted. After the participants had returned to the computer room, they were instructed to play three randomized decision-making games on the computer.

Although participants were told that they would interact with real people in the decision-making games, in actuality their partner was not a person but rather they were partnered with a computer, this allowed maximal experimental control. In order to guarantee the best simulation of a real group decision situation, several measures were adopted in the experiment: (a) At the beginning of the recruitment, all participants were told that they were attending a big experiment that included many participants, and that their participation time in the lab cannot be confirmed until enough participants signed for the same time. (b) All participants remained anonymous during the whole experiment, and the software was programmed that all participants cannot move to the next game until everyone has decided in the current game. (c) To avoid the in-group affiliation (Steinbeis et al., 2015) or the “misery loves company” effect (von Dawans et al., 2012), the instruction booklet indicated clearly that all participants were interacting with strangers in other rooms in the same building. To further win the trust of participants, the software programmed that after all participants made their decisions, there would be an extra random waiting time (2–5 s). (d) To avoid the influence of previous feedbacks on participants’ next decisions, we convinced participants that their feedback would be provided at the end of the experiment. Participants were debriefed and explained about the fake partners after the decision-making games. As a manipulation check, they were asked to fill in the following question individually in their computers: “Did you make your decisions the way you would make them in real life?” Participants who answered “no” to this question, or expressed doubts about the procedures or indicated that they had deliberately adopted a strategy specifically due to the experimental situations were subsequently excluded. Saliva samples were collected at baseline (−25 min), before the TSST-G phase 2 (0 min), in between the TSST-G phase 2 and phase 3 (+10 min), directly after the TSST-G (+20 min), and 15 min, 30 min and 60 min after the stressor (+35, +50, +80 min). Each time point represents the exact time when the participants provided their saliva. After the final salivary sample collection, participants were thanked for their participation and dismissed.

### Measures

#### Empathic Concern Level

Participants’ EC was measured using the EC subscale from Davis’ 28-item Interpersonal Reactivity Index (IRI; Davis, 1983; German version see Huetter et al., 2016). The scale consists of seven items that were answered on a 5-point Likert scale ranging from 0 “never” to 4 “always.” Cronbach’s α for the items were 0.80.

#### Chronic Stress, Major Depression, and Stress Reactivity

To avoid any possible influence from chronic stress, participants filled out the Screening Scale for the Assessment of Chronic Stress (SSCS; Schulz et al., 2004). Cronbach’s α for the items was 0.93. The Patient Health Questionnaire (PHQ-9; Kroenke et al., 2001; German version by Löwe et al., 2002) was used for the measurement of major depression. Cronbach’s α for the items was 0.86. The Perceived Stress Reactivity Scale (PSRS, Schlotz et al., 2011) was applied for the assessment of stress reactivity. Cronbach’s α for the items was 0.91.

#### Subjective Stress Levels

To assess psychologically subjective stress levels, participants indicated their perceived stress level on a 100-mm visual analog scale (VAS). Participants reported their subjective stress levels when they were preparing saliva for cortisol sample collection (seven time points), and besides, they also indicated their subjective stress level after the introduction to the decision-making games (−10 min).

#### Salivary Cortisol

Studies have demonstrated the efficacy of salivary cortisol as a biomarker of HPA axis activity (Kirschbaum and Hellhammer, 1994). For saliva sample collection, participants were instructed to stop swallowing saliva for 2 min until they were asked to transfer the saliva sample into a pre-labeled tube *via* a straw (SaliCap^®^ system; IBL, Hamburg, Germany). All samples were frozen at −20°C for further bio-analyses. When all the behavioral experiments were finished, salivary cortisol was determined by a trained lab assistant using a commercial chemiluminescence immunoassay (ELISA; IBL, Hamburg, Germany) in the Biochemical Laboratory of the Department of Clinical Biopsychology, University of Marburg. The interassay coefficient of variation was 8.48%, and the intraassay coefficient of variation was 11.05%.

### Statistical Analysis

Of all 61 participants, eight scored above 10 points on the PHQ-9 scale, which indicates a high possibility of major depression. Although there was no significant difference in PHQ-9 scores between the control group and the stress group, we decided to include major depression as a covariate for all of the analyses of behavioral and physiological data.

Two-way analyses of covariance (ANCOVAs) for repeated measures with controlling for major depression (PHQ-9) were computed to detect the possible condition (stress, control) × time (repeated factor: eight for subjective stress level and seven for salivary cortisol) interaction effect on subjective stress level and cortisol data. When the requirement of sphericity is violated, the *p*-values were corrected according to Greenhouse-Geisser. To reveal the possible difference between different time points for both the stress groups and the control groups, and also the possible difference between the two groups at a single time point, we used the Bonferroni corrected *post hoc* comparisons, and reported the corrected *p*-values. This method multiplied the uncorrected *p*-values with the number of comparisons and thus the corrected *p*-values can still be evaluated with the value of 0.05. Delta scores were computed by the difference between peak and baseline [delta score of subjective stress level: t4 (+10 min) minus t1 (−25 min); delta cortisol: t6 (+35 min) minus t1 (−25 min)], as indicators of the change of participants’ subjective stress level and stress-induced cortisol.

The main effects of condition on participants’ generosity and the 2PP behaviors were analyzed using one-way ANCOVA, controlling for major depression. Delta cortisol was a continuous variable, and thus linear regression analyses were applied to detect the main effects of it on participants’ generosity and 2PP behaviors. Hierarchical linear regression (HLR) was used when the moderating influence of sex or EC were analyzed. In Step 1, the dependent variable (the prosocial decision-making behavior) was regressed on major depression, which was entered as the control variable. A targeted independent variable (condition/delta-cortisol) and a moderating variable (sex/EC) were subsequently entered as predictors in Step 2. The interaction between the independent variable and the moderating variable was entered in Step 3. Significant interaction effects were further interpreted by simple slope tests. All of the above analyses used SPSS 23.0 software and the PROCESS macro developed by Hayes (2017).

In the strategy method of the 3PC game, participants were presented with six different possible offers from player A, which varied from 0% to 50%. Empirical studies have shown that the proposers and responders usually perceive 40%–50% of the money as a “fair” offer (Hoffman et al., 1996; Jensen et al., 2007). We believed that it was meaningless for a third party to compensate if the situation was perceived as fair, which was supported by our data: 92% of participants refused to compensate if player B was offered 50% of the total money, and 42% refused to compensate player B if the offer was 40% of the total money. Therefore, we only included participants’ possible third-party compensation decisions towards offers above 50% in the analyses. Two-level hierarchical linear modeling (HLM 7, Scientific Software International Inc., Lincolnwood, IL, USA) was conducted with participants’ 3PC behaviors toward different levels of unfair situations (Level 1) nested in subjects (Level 2, controlled for major depression), according to the approach selected by Fehr and Fischbacher (2004). Apart from condition, sex, level of EC, as well as their interactions with condition were considered as Level 2. All continuous variables were standardized (*z*-scored) before computing the interaction items in HLR or HLM models so that multicollinearity issues can be reduced. Participants’ average 3PC behaviors towards offers above 50% were computed before Spearman rank correlations were adopted to verify the association between the three different social-decision behaviors.

## Results

### Demographic Variables and Preliminary Analyses

The control groups and the stress groups did not significantly differ in sex (Chi-square = 0.09, df = 1, *p* > 0.05), EC levels (*t*_(59)_ = 0.28, *p* > 0.05), age (*t*_(59)_ = −1.39, *p* > 0.05), BMI (*t*_(59)_ = −1.44, *p* > 0.05), major depression (*t*_(59)_ = 0.70, *p* > 0.05), dispositional stress reactivity (*t*_(59)_ = 1.83, *p* > 0.05), or chronic stress levels (standard *T*-values, *t*_(59)_ = 0.46, *p* > 0.05, see Table 1).

**Table 1:** Sample statistics.

	Control group (*n* = 30) Mean (SD)	Stress group (*n* = 31) Mean (SD)
Sex	10 men, 20 women	17 men, 14 women
Empathic concern level	2.70 (0.61)	2.66 (0.54)
Age	22.93 (2.91)	24.06 (3.42)
BMI	21.97 (2.61)	23.01 (3.00)
Major depression (PHQ-9)	6.53 (4.95)	5.68 (4.65)
Dispositional stress reactivity (PSRS)	24.47 (8.48)	20.29 (9.35)
Chronic stress levels (SSCS(t))	59.43 (9.21)	58.35 (9.00)

*Note. Total sample *N* = 61. BMI, Body mass index; PHQ-9, The Patient Health Questionnaire; PSRS, The Perceived Stress Reactivity Scale; SSCS(t), The Screening Scale for the Assessment of Chronic Stress (standard *T*-values)*.

### Manipulation Check

**Figure 2:**
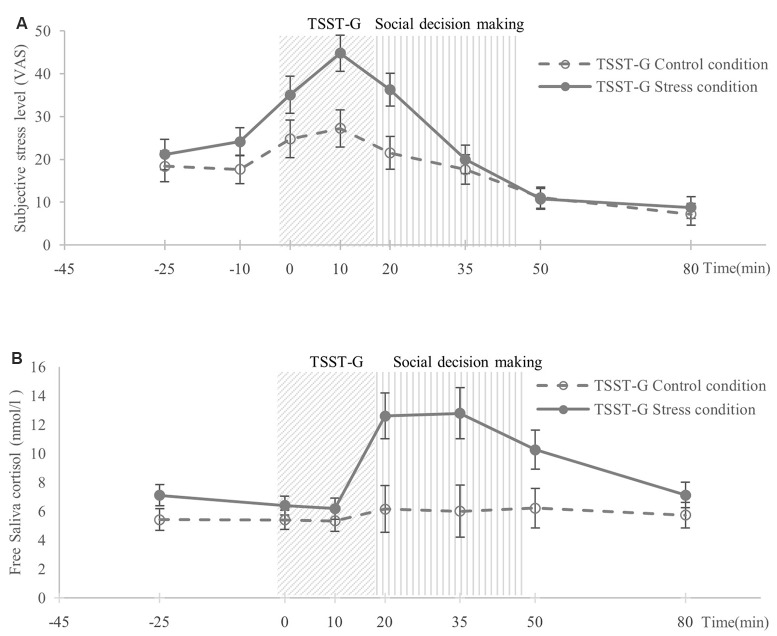
Participants’ **(A)** mean level of subjective stress, **(B)** mean level of salivary cortisol. Time 0 was the start of the Trier Social Stress Test for Groups (TSST-G; von Dawans et al., 2011). Error bars indicate standard errors of the mean.

The analysis of subjective stress level (VAS) revealed significant effects of time (*F*_(3.94,228.62)_ = 8.02, *p* < 0.001, *η*^2^ = 0.12), trend-level effects of condition (*F*_(1,58)_ = 2.98, *p* = 0.09, *η*^2^ = 0.05), and a significant condition × time interaction (*F*_(3.94,228.62)_ = 4.28, *p* < 0.01, *η*^2^ = 0.07). Bonferroni corrected pairwise comparisons showed that in the stress condition, subjective stress level increased immediately after exposure to the TSST-G [t3(+0 min)], and the high level remained until 20 min after the exposure [t5(+20 min)]. The subjective stress levels of these time points were all significantly higher than the baseline t1 (all *p*s < 0.01). In contrast, in the control condition, subjective stress levels at the time points from t3 to t5 did not differ from the baseline (all *p*s > 0.05). Furthermore, the subjective stress ratings at t4 (+10 min) and t5 (+20 min) were significantly higher in the stress condition than in the control condition (both *p*s < 0.01; Figure 2A).

The analysis of cortisol level indicated significant effects of time (*F*_(2.47,143.13)_ = 3.28, *p* < 0.05, *η*^2^ = 0.05), significant effects of condition (*F*_(1,58)_ = 6.45, *p* < 0.05, *η*^2^ = 0.10), and a significant condition × time interaction (*F*_(2.47,143.13)_ = 4.49, *p* < 0.01, *η*^2^ = 0.07). Bonferroni corrected pairwise comparisons revealed that in the stress condition, salivary cortisol level increased 20 mins after exposure to the TSST-G (t5), and continued to 35 min after the exposure (t6). Cortisol levels at both time points were significantly higher than the baseline (t1; both *p*s < 0.01). As to the control condition, cortisol levels at all time points remained non-significant compared with the baseline (t1; all *p*s > 0.05). Moreover, when compared with cortisol levels in the control group, the cortisol levels in the stress groups were significantly higher at t5 (+20 min; *p* < 0.01), t6 (+35 min; *p* < 0.01), and t7 (+50 min; *p* < 0.05; Figure 2B).

Significant effects of condition in delta scores of subjective stress (*F*_(1,58)_ = 7.20, *p* < 0.01, *η*^2^ = 0.11) and in delta scores of cortisol level (*F*_(1,58)_ = 4.10, *p* < 0.05, *η*^2^ = 0.07) also emerged. Thus, the TSST-G stress manipulation successfully induced both psychological and physiological responses.

Participants played both the dictator game and the ultimatum game twice, once as a responder and once as a proposer. The sequences of their roles in the dictator game did not influence participants’ generous behaviors (*F*_(1,58)_ = 1.44, *p* > 0.05), and its interaction with the condition was also not significant (*F*_(1,56)_ = 1.07, *p* > 0.05). The sequences of their roles in the ultimatum game also did not influence participants’ 2PP behaviors (*F*_(1,58)_ = 0.05, *p* > 0.05), and its interaction with the condition was also not significant (*F*_(1,56)_ = 2.18,*p* > 0.05).

### Main Effects of Stress Condition and Cortisol Reactivity

There was no significant main effect of condition on generosity in the dictator game (*F*_(1,58)_ = 0.28, *p* > 0.05), 2PP behavior in the ultimatum game (*F*_(1,58)_ = 0.26, *p* > 0.05), or 3PC behavior (*F*_(1,58)_ = 0.30, *p* > 0.05).

A significant effect of delta cortisol on 3PC behavior emerged (*t*_(58)_ = 2.16, *p* < 0.05, *r*^2^ = 0.01), however, there was no significant main effect of delta cortisol on generosity in the dictator game (*F*_(1,58)_ = 0.07, *p* > 0.05) or 2PP behavior in the ultimatum game (*F*_(1,58)_ = 2.77, *p* > 0.05). Major depression (PHQ-9 score) as a covariate was not significant in any of the analyses.

Spearman rank correlations found significant association between participants’ generosity and their average 3PC behaviors (*r*_s_ = 0.49, *p* < 0.001), but no association between generosity and 2PP behaviors (*r*_s_ = −0.01, *p* > 0.05), or 2PP behaviors and 3PC behaviors (*r*_s_ = 0.14, *p* > 0.05).

### Sex-Specific Effects

There was no sex difference on delta cortisol (*F*_(1,58)_ = 0.61, *p* > 0.05) and subjective stress (*F*_(1,58)_ = 0.08, *p* > 0.05), and also no interactive effect of sex and condition on delta cortisol (*F*_(1,56)_ = 0.06, *p* > 0.05), and on subjective stress (*F*_(1,56)_ = 0.21, *p* > 0.05).

**Figure 3:**
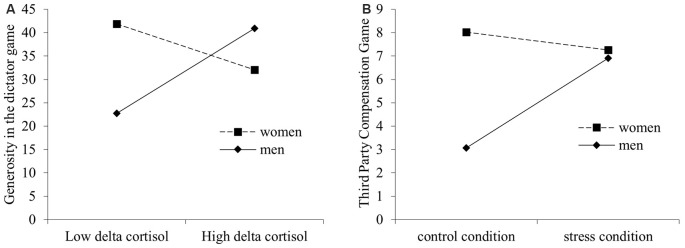
Simple slope plots of **(A)** cortisol-delta × sex interactive effects on participants’ generosity in the dictator game and **(B)** condition × sex interaction effect on participants’ third-party compensation behaviors. Low = Mean − 1 SD; High = Mean + 1 SD.

Results found no significant interactive effect of sex and condition on generosity in the dictator game (*F*_(1,56)_ = 2.50, *p* > 0.05), however, a significant interactive effect of sex and delta cortisol (ΔR^2^ = 0.10, *F*_(1,56)_ = 6.40, *p* < 0.05) emerged (see Table 2). The significant interactive effect was interpreted using simple slope analysis, which revealed that cortisol increase did not influence women’s generosity in the dictator game (*B* = −4.90, *p* > 0.05), but that men showed more generosity in the dictator game (*B* = 9.07, *p* < 0.05) as cortisol increased (Figure 3A).

**Table 2:** Hierarchical linear regression analysis for delta cortisol and sex on generosity.

	Step 1 *B (SE)*	Step 2 *B (SE)*	Step 3 *B (SE)*
Intercept	35.43*** (2.70)	37.38*** (3.67)	36.99*** (3.51)
Major depression	1.22 (2.72)	1.02 (2.77)	−0.62 (2.72)
Delta cortisol		0.94 (2.77)	−4.90 (3.51)
Sex		−4.41 (5.55)	−5.15 (5.31)
Delta cortisol × Sex			13.97* (5.52)
*R^2^*	−0.01	−0.04	0.05
ΔR^2^	0.003	0.01	0.10
*F*	0.20	0.35	6.40*

Note. Total sample N = 61. Binary variable sex was coded 0 = female, 1 = male. The control variable major depression and independent variable delta cortisol were standardized prior to computing interaction terms. *B* = unstandardized regression coefficient. *SE* = standard error (of *B*). *p < 0.05, ***p < 0.001.

The interaction between sex and condition on 3PC behavior was found not significant but with a trend effect (*t*_(56)_ = 1.77, *p* = 0.08, *r*^2^ = 0.03, see Table 3). To test our initial hypothesis regarding sex difference, we nevertheless conducted a simple slope analysis, which showed that the women’s 3PC was not influenced by stress condition (*B* = −0.75, *p* > 0.05); in contrast, men showed higher 3PC behavior in the stress group compared to the control group (*B* = 3.84, *p* < 0.05; Figure 3B). However, we did not find a significant interactive effect of sex and cortisol reactivity on 3PC behavior (*t*_(56)_ = 0.54, *p* > 0.05).

**Table 3:** Hierarchical linear model for condition and sex on third party compensation behaviors.

	*B*	*SE*	*t*	*p*
**Level 1**
Unfair levels	0.15	0.02	6.03	<0.001
**Level 2**
Intercept	8.02	0.98	8.19	<0.001
Major depression	−0.19	0.63	−0.30	0.77
Condition	−0.75	1.68	−0.44	0.66
Sex	−4.95	1.47	−3.36	0.001
Condition × Sex	4.59	2.60	1.77	0.08

*Note. Total sample *N* = 61. Binary variable condition was coded 0 = control group, 1 = stress group; sex was coded 0 = female, 1 = male. The control variable major depression was standardized and the control variable unfair levels was grand centered before entered into model. *B* = unstandardized regression coefficient. *SE* = standard error (of *B*)*.

There was no significant interactive effect of sex and condition on 2PP behavior (*F*_(1,56)_ = 0.82, *p* > 0.05), and no significant interactive effect of sex and cortisol reactivity on 2PP behavior (*F*_(1,56)_ = 0.19, *p* > 0.05). Major depression (PHQ-9) as a covariate was not significant in any of the analyses.

### The Moderating Effect of Empathic Concern

**Figure 4:**
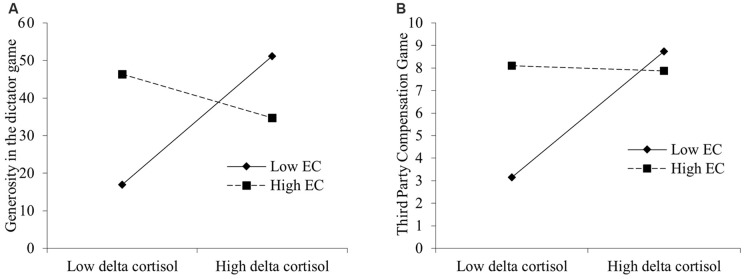
Simple slope plots of cortisol-delta × EC interactive effects on participants’ **(A)** generosity in the dictator game, and **(B)** third-party compensation behaviors. EC, Empathic Concern. Low = Mean − 1 SD; High = Mean + 1 SD.

While EC did not predict any of the three prosocial behaviors (all *p*s > 0.05), there was no significant interactive effect of EC and condition on the generosity in the dictator game (*F*_(1,56)_ = 1.90, *p* > 0.05), the 2PP behavior (*F*_(1,56)_ = 3.01, *p* > 0.05) and the 3PC behavior (*t*_(56)_ = −0.52, *p* > 0.05). Further analyses indicated that the interactive effect of EC and delta cortisol on generosity in the dictator game reached significant (ΔR^2^ = 0.15, *F*_(1,56)_ = 10.18, *p* < 0.01, see Table 4), and that the interactive effect on 3PC behavior was significant on a trend level (*t*_(56)_ = −1.92, *p* = 0.06, see Table 5). As revealed by simple slope analysis, participants with low EC reported more generosity in the dictator game (*B* = 17.08, *p* < 0.01; Figure 4A) and more 3PP behaviors (*B* = 2.79, *p* < 0.05; Figure 4B) as cortisol increased. In contrast, the generosity (*B* = −5.83, *p* > 0.05; Figure 4A) and 3PP behaviors (*B* = −0.12, *p* > 0.05; Figure 4B) of participants with high EC were not influenced by cortisol reactivity. There was no significant interactive effect of EC and cortisol reactivity on the 2PP behaviors (*F*_(1,56)_ = 1.20, *p* > 0.05). Major depression (PHQ-9) as a covariate was not significant in any of the analyses. Women reported significantly higher EC level, compared to men (women: *M* = 2.87, SD = 0.47; men: *M* = 2.44, SD = 0.61; *t*_(59)_ = 3.04, *p* < 0.01, *d* = 0.80).

**Table 4:** Hierarchical linear regression analysis for delta cortisol and empathic concern on generosity.

	Step 1 *B (SE)*	Step 2 *B (SE)*	Step 3 *B (SE)*
Intercept	35.43*** (2.70)	35.43*** (2.68)	37.34*** (2.56)
Major depression	1.22 (2.72)	1.44 (2.70)	−1.44 (2.67)
Delta cortisol		−0.06 (2.74)	5.63 (3.11)
EC		4.65 (2.74)	3.25 (2.58)
Delta cortisol × EC			−11.46** (3.59)
*R*^2^	−0.01	0.003	0.14
ΔR^2^	0.003	0.49	0.15
*F*	0.20	1.48	10.18**

Note. Total sample *N* = 61. The control variable major depression, independent variable delta cortisol and moderator variable empathic concern (EC) were standardized prior to computing interaction terms. *B* = unstandardized regression coefficient. *SE* = standard error (of *B*). **p < 0.01, ***p < 0.001.

**Table 5:** Hierarchical linear model for delta cortisol and empathic concern on third party compensation behaviors.

	*B*	*SE*	*t*	*p*
**Level 1**
Unfair levels	0.15	0.02	6.03	<0.001
**Level 2**
Intercept	6.97	0.63	10.97	<0.001
Major depression	−0.30	0.71	−0.41	0.68
Delta cortisol	1.33	0.50	2.67	0.01
EC	1.02	0.62	1.64	0.11
Delta cortisol × EC	−1.45	0.76	−1.92	0.06

*Note. Total sample *N* = 61. The control variable major depression, independent variable delta cortisol and moderator variable empathic concern (EC) were standardized prior to computing interaction terms. B = unstandardized regression coefficient. SE = standard error (of B)*.

## Discussion

In the present article, we reported findings of an experimental study examining sex differences in participants’ prosocial decision-making behaviors following acute stress. As most of the previous research focused on the mere sex differences between stress group and control group, this is the first study to further investigate the potential sex-specific differences in the impact of cortisol reactivity on prosocial decision-making. We have found evidence for the interactive effects of sex and cortisol reactivity on participants’ generosity, that men were more generous as their cortisol reactivity increased. We also observed a trend effect that men in the stress group reported more 3PC behaviors compared to men in the control group. To our surprise, we did not observe such responses in our female participants in any of the social decision-making tasks.

These findings are in keeping with previous research, which revealed that men had a tend-and-befriend tendency under stress (e.g., von Dawans et al., 2012; Sollberger et al., 2016), or a positive association between the cortisol response and men’s prosocial behaviors (e.g., Berger et al., 2016; Sollberger et al., 2016; Singer et al., 2017; Margittai et al., 2018). However, our data contrast with other research which linked stress or cortisol reactivity with individuals’ selfishness, sense of competition and less generosity (Vinkers et al., 2013; Nickels et al., 2017).

One possible explanation may lie in the prior social cue which was uniquely provided in the TSST-G protocol. Minimal social cues have been proved to have a salient impact on participants’ generosity, but only among male participants (Rigdon et al., 2009). It is reasonable to interpret that the group protocol of TSST-G provided such social cues that prime men’s prosocial orientation in the upcoming tasks. Congruously, several studies adopting the TSST-G paradigm also reported tend-and-befriend responses of men in stress situations (von Dawans et al., 2012; Margittai et al., 2015), or men with a high level of cortisol reactivity (Berger et al., 2016). Human beings are social mammals with community habits, and thus they have to make most decisions in social contexts. Therefore, the TSST-G protocol is more suitable and has a better ecological validity compared to the TSST when individuals’ subsequent decision-making behaviors are considered. Furthermore, the TSST-G paradigm could provide enough participants for the subsequent social tasks which involved interactions with multiple persons, and thus has been frequently used in studies related to social behaviors (e.g., von Dawans et al., 2012, 2019; Margittai et al., 2015; Berger et al., 2016). As for the female participants in our study, they may have a lower sensitivity to social cues compared to male participants, and thus were not influenced by the stress condition or by cortisol elevation. Future studies focusing on participants’ prosocial behaviors after acute stress should also explicitly consider the impact of social context.

The inconsistent effects of stress condition and cortisol reactivity on men’s different prosocial behaviors may lie in the frame of the social tasks, and pointed out the conditional tend-and-befriend responses under stress. Men’s generosity and 3PC behavior were reported to have positive associations with stress or cortisol reactivity, however, the 2PP behavior had no associations with stress or stress-related cortisol change, in line with previous research (von Dawans et al., 2012; Nickels et al., 2017). The frames of these behaviors may interpret the inconsistency: the generous division in the dictator game and 3PC behavior were framed as positive reactions towards others. Nevertheless, the 2PP behavior in the ultimatum was framed as a negative reaction with the goal of reducing other’s payment (Pfattheicher and Keller, 2014), even though the 2PP behavior was driven by a prosocial motivation of norm enforcement (Fehr and Gächter, 2002). Generally, social decision-making has emphasized the dual-process systems: an impulsive system that processes information automatically, unconsciously and effortlessly regarding cognitive resources; and a reflective system that processes information in a controlled, conscious and effortful way (Strack and Deutsch, 2004, 2012). Since stress altered the balance of the dual systems and enhances the impulsive system with less cognitive resources (Finy et al., 2014; Lu et al., 2014), it is possible that stressed men only processed the frame information of the 2PP behavior and ignored the altruistic consequences. As a matter of fact, framing effects are the demonstration that the impulsive system prevails over the reflective system (Kahneman and Frederick, 2007). In addition, we have discovered a significant association between participants’ generosity and 3PC behaviors, but not between 2PP behaviors and the other two behaviors, which also pointed out that the three behaviors are perceived and processed differently. Collectively, stress or more specifically stress-related cortisol elevation enhances men’s prosocial decision-making, but only decisions with positive frames which lead to direct prosocial consequences of others. Future studies are expected to consider the frame of prosocial behaviors empirically.

The fact that the men’s cortisol elevation positively predicted both men’s generosity and 3PC behaviors revealed that cortisol reactivity could be a main mechanism beneath men’s tend-and-befriend responses towards stress condition. It is notable that we only observed the effect of stress on men’s 3PC behavior, but not on their generosity in the dictator game as the previous scientists did (von Dawans et al., 2012). On one hand, the two parties in the dictator game are bonded in their economic payments that whenever someone earned more, the other one earned less, while the direct connection of interests was prevented by the third-party compensation game, and allow participants to make a prosocial decision based on their norm enforcement (Leliveld et al., 2012). Therefore, it is possible that the prosocial motivation of 3PC behaviors are stronger than the generous divisions in the dictator game, and are more sensitive to the situational stress. Although, maybe a larger sample size may shed more light on this inconsistency. In short, prosocial decision-making behaviors may be more sensitive to the change of cortisol, compared to the mere exposure to a stressor.

The current study observed the significant effect of cortisol reactivity on 3PC behaviors among both genders, but the impact of stress on women’s 3PC behaviors was not significant. Apart from this, women’s decision-making behaviors were not associated with stress condition or stress-related cortisol, which is at odds with previous research (Smeets et al., 2009; Tomova et al., 2014; von Dawans et al., 2019). In fact, the present study has observed a consistent pattern in the role of sex and EC, namely, the prosocial behaviors of men and participants with low EC increased as stress-related cortisol elevated, while the prosocial behavior of women and participants with high EC were unaffected. Simultaneously, the female participants reported higher EC than males. As a result, our preliminary speculation is that women’s prosocial behaviors remained unchanged across stress condition because of their EC level, and EC could be an alternative moderator to sex, to distinguish individuals’ stress responses, due to its unique social feature. This part would be discussed in a later paragraph related to EC. Future research is also expected to disentangle the reasons behind the difference between the current research and the previous literature. Moreover, future studies should also consider the possibility that women’s prosocial decision-making behaviors under stress condition may be predicted by other stress systems, such as the ANS systems indicated by salivary α-amylase and heart rate.

The results for EC are generally consistent with our predictions, given the integration of the state-dependent and the trait-consistent hypotheses, that EC moderated the effect of cortisol elevation on prosocial decision-making behaviors following acute stress, with the exception of the 2PP behaviors. Specifically, participants with low EC tended to allocate more money to their partners in the dictator game and were more willing to engage in 3PC behavior towards victims who suffered unfair treatment, following cortisol elevation. By contrast, participants with high EC were not influenced by the cortisol elevation in any of these social tasks. These results may be further explained, concerning EC and prosocial sensitivity. Following the trait-dependent theory, high-EC individuals are generally more sensitive to the feelings of others, and thus are persistent to react with tend-and-befriend responses across different situations (Davis, 1983; Leliveld et al., 2012). Oppositely, low-EC individuals are less disposed to exhibit prosocial decisions until the stress-induced cortisol elevation intervenes. Wolf et al. (2015) have shown that acute stress could enhance emotional empathy in healthy young men. Although they did not include both genders and considered the trait differences of the EC level, the change in emotional empathy due to stress may explain the current findings of low-EC individuals. Simultaneous measurement of empathy and social decision-making after the stressor is a promising approach for future investigations.

In addition, the results indicated that the dispositional variable EC might be an alternative moderator to sex, to distinguish individuals with tend-and-befriend responses as cortisol increases. Interestingly, women in the current study reported significantly higher EC levels compared with men, and the behavioral patterns of men are very similar to low-EC individuals. Scientists had heated debates about the different behavioral stress responses from the perspective of the evolution (Cannon, 1932; Taylor et al., 2000; Geary and Flinn, 2002), and tried to understand the fight-or-flight responses and tend-and-befriend responses in terms of the physiological sex difference. In this current study, we proposed an alternative perspective that we could pay more attention to the social nature of human, and considered the unique social traits humans developed in the long history of evolution. It will be beneficial in future research to investigate the similar role of sex and EC in modulating the impact of cortisol reactivity on prosocial decision-making behaviors. Moreover, considering sex from a social or cultural perspective, such as gender identity and gender socialization, could also shed new light on research related to the sex-effect of stress responses. Further studies are suggested to take this into account, along with considering gender roles other than a merely binary option (Dedovic et al., 2009; Alvarado et al., 2018).

This study has several strengths that need to be acknowledged. The first advantage of the study is that we included both genders in the experiment, and thus comprehensively examined the impact of stress and stress-related cortisol change on prosocial behaviors. To our knowledge, this is the first study to focus on sex differences in the impact of cortisol reactivity on prosocial decision-making behaviors. Second, the TSST-G was employed as the stressor and successfully induced physiological and psychological stress. We believed that the protocol is a better simulation of the real social decision context, and has provided social cues that prime men’s prosocial orientation. Therefore, we suggest that it should be used more frequently in future stress studies related to social tasks. Third, the study was the first to test the essential empathy trait that affects human’s social behaviors in this context, and we demonstrated that EC could distinguish individuals who exhibited tend-and-befriend responses as cortisol increases towards acute stress.

Nevertheless, several limitations also have to be considered. First of all, one could argue that the best experimental design to examine the impact of cortisol elevation would be the employment of exogenous administration of glucocorticoids (GCs). However, the generalizability from such experiments to a real-life situation can be called into question. In terms of the social tasks assessed following the stressor, we decided to adopt the TSST-G task and measured the naturally released hormones from the human body. A second limitation lies in the size and distribution of the sample. To control for the impact of individual differences due to heterogeneity, and to avoid any unexpected influence on stress measurement, we screened and invited only medically healthy students to the lab and strictly controlled their physical activities before their arrival. As such, the final sample size was reduced. Even though the number has met the minimum requirement to reach a reasonable conclusion, the number of participants in each group is different. Due to the difficulty of organizing spontaneous experiments involving four participants, the sex ratio of each four-person group depends on the schedule of the participants. Since the experimental condition of each four-person group is randomly determined, a gender imbalance eventually occurred in the control group and stress group. Although the statistical analyses demonstrated the difference has not reached significant, a larger sample size with a fairer distribution would be suggested in the future study. Thirdly, it would be advisable for further research to broaden the sample to a more general population (not only a student sample) and include a greater diversity of participants, such as patients with stress-related diseases. Next, we were not able to control the menstrual cycle in women. However, given the potential impact of sex hormone fluctuations over the course of the cycle, future studies should examine the impact of menstrual cycle phase on stress-related prosocial decisions (Childs et al., 2010). Finally, other factors that might have been of potential importance to our targeted variables were not included in our research and future research would need to take a closer look at them. For instance, potential psychiatric and medical comorbidities could be assessed *via* standardized clinical interviews and/or medical examinations, as these factors have the potential to influence cortisol regulation (Almela et al., 2011). Similarly, it might be reasonable to control for levels of the hormone oxytocin as it has been previously shown to be involved in human prosocial behavior (Barraza and Zak, 2009). Moreover, some factors that might also be related to sex such as occupation, marital status, education, or SEM could also have an impact on individuals’ prosocial behaviors and should be considered in future studies.

## Conclusion

In summary, the first aim of the present study was to determine the moderating role of sex in the association between the stress-induced cortisol and prosocial decision-making behaviors immediately after stress. This aim was exploratory due to the mixed findings from previous literature (e.g., von Dawans et al., 2012; Vinkers et al., 2013; Nickels et al., 2017). The second aim was to examine the moderating role of EC in the association between the stress-induced cortisol and prosocial decision-making behaviors immediately after stress, and we hypothesized that stress and stress-related cortisol elevation were more predictive on decision-making behavior at a low level of EC rather than on the high level of EC. Overall, we found that men in the stress group showed more 3PC behaviors compared in the control group, and the magnitude of cortisol secretion successfully predicted men’s generosity and 3PC behaviors. These findings suggested that men exhibited “tend-and-befriend” responses to stressful situations especially when their HPA axis is highly activated. Moreover, EC as a predictive dispositional variable was found to moderate the effect of stress-related cortisol responses on prosocial decision-making behaviors following acute stress. In line with the integration of the state-dependent and the trait-consistent hypotheses, low-EC individuals reported enhanced generosity and 3PC behaviors as their cortisol elevated, while high-EC individuals’ prosocial decision-making behaviors remained unchanged across different situations. Therefore, we suggested that EC as an alternative moderator should be paid more attention when we discuss social decision-making under acute stress. In sum, the present study contributes to a better comprehension of the behavioral stress responses and points out the essential moderating role of sex and EC.

## Data Availability Statement

The datasets generated for this study will not be made publicly available because we do not have permissions from the participants to share the data. Requests to access the datasets should be directed to the corresponding author.

## Ethics Statement

The studies involving human participants were reviewed and approved by the local Ethics Committee of Department of Psychology, University of Marburg, Germany. The participants provided their written informed consent to participate in this study.

## Author Contributions

QZ, JM and UN contributed to the study design, data analysis and interpretation were performed. Testing and data collection were performed by QZ and UN. QZ drafted the manuscript, and JM and UN provided critical revisions. All authors approved the final version of the manuscript for submission.

## Conflict of Interest

The authors declare that the research was conducted in the absence of any commercial or financial relationships that could be construed as a potential conflict of interest.
